# Monoclonal Antibody Targeting Neutralizing Epitope on H5N1 Influenza Virus of Clade 1 and 0 for Specific H5 Quantification

**DOI:** 10.1155/2013/360675

**Published:** 2013-03-05

**Authors:** Fang He, Jimmy Kwang

**Affiliations:** ^1^Animal Health Biotechnology, Temasek Life Sciences Laboratory, National University of Singapore, Singapore 117604; ^2^Department of Microbiology, Faculty of Medicine, National University of Singapore, Singapore 117597

## Abstract

H5N1 influenza viruses cause high mortality in avian and mammalian species, including humans. Antigenic drift in H5 sequence poses challenges in the development of vaccine and therapeutic antibody. In this study, a monoclonal antibody 11G12 was produced from inactivated H5N1 immunized mice. Results from IFA, ELISA, HI, and virus neutralization indicated that Mab 11G12 can specifically recognize and neutralize H5 type hemagglutinin from clade 1 and 0 without any cross-reaction to any other clades of H5N1 viruses. Mab 11G12 was used to differentiate and quantify the expression of H5N1 strain A/VietNam/1203/04 from a trivalent vaccine mix in ELISA. Sequencing of escape mutants identified that Mab 11G12 targets a major neutralizing epitope of influenza H5 hemagglutinin. The study indicated that some major neutralizing epitopes in H5s of early strains were mutated due to antigenic drift.

## 1. Background

Highly pathogenic avian influenza H5N1 virus has caused high mortality in birds and humans, raising concerns for the possibility of a future influenza pandemic [1]. In 1997, in Hong Kong, 18 humans were infected and 6 died in the first known case of H5N1 infecting humans [2, 3]. Since the 2004 outbreaks of H5N1 influenza viruses from birds to human in Vietnam and Thailand, newly emerging avian influenza A viruses pose a continued lethal threat, not only to avian species but also to humans [4, 5]. The H5N1 influenza viruses are currently divisible into 10 clades (0 to 9) on the basis of phylogenetic analysis of their hemagglutinin (HA) genes that have evolved in the A/Goose/Guangdong/96-like H5N1 lineage (Clade 0) [6]. Clade 0 includes all the early progenitors which are predominately strains in 1996–2002 from Hong Kong (HK) and China, while clade 1 includes human and bird isolates from Vietnam, Thailand, and Cambodia and bird isolates from Laos and Malaysia [7]. The human isolate A/VietNam/1203/04 (H5N1) from the 2004 outbreak was identified as the most pathogenic isolate in Clade 1.0 [8, 9]. It was widely selected as the vaccine strain for H5N1. The stockpiling of a panel of vaccines with hemagglutinin (HA) antigenic variations, including A/VietNam/1203/2004, A/VietNam/1194/2004, A/Indonesia/05/2005, and A/Anhui/1/2005 vaccine viruses, was recommended by the WHO for vaccine development [7]. 

However, present vaccine strategies have been hindered by antigenic variation of the influenza strains. Immunity elicited with a single strain from a previous outbreak may not be able to provide sufficient protection against currently circulating H5N1 viruses [10]. Preventive measures against circulating H5N1 strains have received a lot of interest and effort globally to prevent another pandemic outbreak [11]. To overcome such limitations without any reduction in the vaccine efficacy and to completely realize the potential of vaccines worldwide, the concept of universal vaccines based on representative hemagglutinin mix has recently been proposed [12]. With an understanding in the distribution pattern of HA neutralizing epitopes, a trivalent H5 vaccine has been developed and proved to provide universal protection against multiple clades of H5 influenza viruses. The trivalent formulation includes H5N1 strains A/Indonesia/CDC669/2006 (clade 2.1), A/VietNam/1203/2004 (clade 1), and A/Anhui/1/05 (clade 2.3) [13]. In order to specifically and individually identify and quantify HA expression of each strain, several strain-specific H5 monoclonal antibodies were selected and characterized (data not shown). In the present study, a monoclonal antibody 11G12 can specifically interact in IFA with H5 of the VN1203 strain without any cross-activity to either the Indonesia or Anhui strains. The reactivity was further evaluated in ELISA, HI, and virus neutralization against different clades of H5N1 viruses. The antigenic epitope for 11G12 was identified on H5 hemagglutinin. Results indicate that 11G12 is specific to H5N1 of clade 1.0 and 0.

## 2. Materials and Methods

### 2.1. Viruses and Cells

The H5N1 viruses used in these studies are from different clades (clade 0—A/HongKong/156/97, clade 1.0—A/HongKong/213/04, clade 1.0—A/VietNam/1203/04, clade 2.1.3—A/Indonesia/CDC669/06, clade 2.1.2—A/Indonesia/CDC594/06, clade 2.2—A/Nigeria/6e/07, clade 2.2—A/chicken/Guangdong/178/04, clade 2.3—A/Anhui/1/05, clade 2.3—A/Jiangsu/2/07, clade 4—A/goose/Guiyang/337/06, clade 7—A/chicken/Shanxi/2/06 and clade 8—A/chicken/Henan/12/04). Except for the two Indonesian strains, the remaining H5 influenza viruses were generated with reverse genetics in our lab as described previously [14]. Viruses were inoculated into the allantoic cavities of 11-day-old embryonated chicken eggs and harvested following 48 h of incubation at 37°C. Virus titers were determined using hemagglutination assays according to standard methods [15]. H5N1 subtype viruses were inactivated with formaldehyde as described previously [16]. All experiments with live H5N1 subtype viruses were performed in a biosafety level 3 containment laboratory in compliance with CDC/NIH and WHO recommendations and also were approved by the Agri-Food and Veterinary Authority and the Ministry of Health of Singapore.

MDCK cells were obtained from the American Type Culture Collection (ATCC). Cells were propagated in Dulbecco's minimal essential medium (DMEM) supplemented with 10% fetal bovine serum. Virus stocks were grown in MDCK cells in DMEM supplemented with 0.5% bovine serum albumin (BSA) and 200 ng/mL of trypsin.

### 2.2. MAb Production

BALB/c mice were immunized twice subcutaneously at regular intervals of 2 weeks with inactivated whole virus from A/VietNam/1203/2004 at HA titer of 2^8^ in 0.1 mL of Phosphate Buffered Saline (PBS), which was emulsified with an equal volume of Montanide ISA 563 adjuvant (SEPPIC, France). Mice were boosted with the same viral antigen, 3 days before the fusion of splenocytes with SP2/0 cells. The fused cells were seeded in 96-well plates, and their supernatants were screened by immunofluorescence assays as described below. The hybridomas that produced the Mabs were cloned by limiting dilution at least three times. The positive Mabs were tested for their hemagglutination inhibition activity as described below. Immunoglobulins from selected positive Mabs were isotyped using a commercial isotyping kit (Amersham Bioscience, England) as described in the manufacturer's protocol, subcloned, and cultured. All animal experiments were carried out in accordance with the guides for Animal Experiments of the National Institute of Infectious Disease (NIID) and the “Animal Research: Reporting In Vivo Experiments” (ARRIVE) guidelines. Experiment protocols were reviewed and approved by Institutional Animal Care and Use Committee of the Temasek Life Sciences Laboratory (Project Approval nos. TLL-12-018 and TLL-12-015), National University of Singapore, Singapore.

### 2.3. Immunofluorescence Assay (IFA)

MDCK cells cultured in 96-well plates were infected with AIV H5N1 strains. At 24–48 h postinfection, the cells were fixed with 4% paraformaldehyde for 30 min at room temperature and washed thrice with phosphate buffered saline (PBS), pH 7.4. Fixed cells were incubated with hybridoma culture supernatant at 37°C for 1 h, rinsed with phosphate buffered saline (PBS), and then incubated with a 1 : 400 dilution of fluorescein isothiocyanate (FITC)-conjugated rabbit antimouse Immunoglobulin (Dako, Denmark). Cells were rinsed again in PBS, and antibody binding was evaluated by widefield epifluorescence microscopy (Olympus IX71).

### 2.4. Hemagglutination Inhibition Assay

Hemagglutination inhibition (HI) assays were performed as described previously [17]. Briefly, Mabs were serially diluted (2-fold) in V-bottom 96-well plates and mixed with 4 HA units of H5N1 viruses. Plates were incubated for 30 min at room temperature, and 1% chicken RBCs were added to each well. The hemagglutination inhibition endpoint was the highest Mab dilution in which agglutination was not observed.

### 2.5. ELISA

96-well, round-bottom microtiter plates (Nunc, Roskilde, Denmark) were coated with 1 ug/well of capture MAb 11G12 in 100 uL of carbonate buffer (73 mM sodium bicarbonate and 30 mM sodium carbonate, pH 9.7) overnight at 4°C or 37°C for 2 h. The plates were washed twice with PBST, followed by two washes with PBS after each incubation with antibody or antigen. The antibody-coated plates were blocked by incubation with 100 uL of blocking buffer (PBS containing 5% milk) for 1 h at room temperature and then incubated at 37°C for 1 h with 100 uL of virus-containing samples diluted in PBST. Virus binding was detected by incubation for 1 h at 37°C with 100 uL of horseradish peroxidase-conjugated detection MAb 11G12 (800 ng) (in-house labeling; Roche). Chromogen development was mediated by the addition of 100 uL of freshly prepared substrate solution (o-phenylenediamine-dihydrochloride; Sigma). The reaction was stopped by adding 0.1 N sulfuric acid, and the optical density at 490 nm was recorded. The detection limit was determined by the optical density value that gave a signal-to-noise ratio of 3.

### 2.6. Microneutralization Assay

Neutralization activity of the monoclonal antibody against H5N1 strains was analyzed by microneutralization assay as previously described [18]. Briefly, ten times diluted Mab was further serially diluted (2-fold) and incubated with 100 50% tissue culture infectious doses (TCID50) of different clades of H5N1 strains for 1 h at room temperature and plated in duplicate onto MDCK cells grown in a 96-well plate. The TCID50 of each of the H5N1 strains in MDCK cell culture was determined by the Reed and Muench method. The neutralizing titer was assessed as the highest Mab dilution in which no cytopathic effect was observed by light microscopy.

### 2.7. Isolation and Analysis of Escape Mutants

The epitope recognized by Mab 11G12 was mapped by characterization of escape mutants as described previously [19, 20]. Briefly, H5N1 viruses were incubated with an excess of Mab for 1 h and then inoculated into 11-day-old embryonated chicken eggs. The eggs were incubated at 37°C for 24–48 h. Virus was harvested and used for cloning in limiting dilution in embryonated chicken eggs, and the escape mutants were plaque purified. RNA was extracted from the allantoic fluid. The hemagglutinin gene was reverse transcriptase (RT)-PCR, amplified, and cloned into a TA-cloning vector (Promega), and several clones were sequenced. The sequences of individual clones were analyzed by comparison with the sequences of the parent virus.

## 3. Results

### 3.1. Characterization of Murine Monoclonal Antibody 11G12

Mab 11G12 was produced from mice immunized with A/VietNam/1203/2004 H5N1 virus. 11G12 belongs to isotype IgM. 11G12 is able to detect H5 expression in MDCK cells infected with H5N1 strain A/VietNam/1203/2004 (VN1203) in IFA without any cross-reaction with uninfected MDCK cells. It presents neutralizing activity against VN1203 as indicated in HI and virus neutralization test. No activity was observed in Western blot with 11G12 against any H5 protein. All these findings suggest that Mab 11G12 recognizes a neutralizing epitope in H5 of VN1203.

### 3.2. Specific Interaction with H5 Viruses in Clade 0 and 1 with 11G12

**Figure 1:**
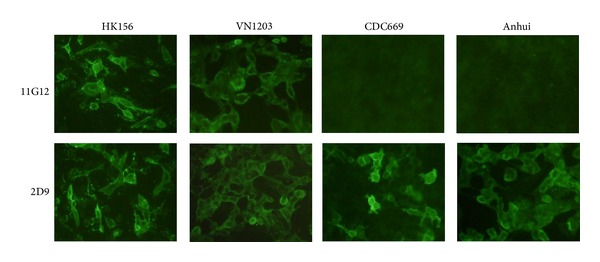
Reactivity against different H5 strains with 11G12 in H5 infected MDCK cells with IFA. MDCK cells were infected with H5 influenza virus as listed. Fixed cells were stained with either 11G12 or 2D9. 2D9 is a Mab with broad recognition to all these H5 strains used.

**Figure 2:**
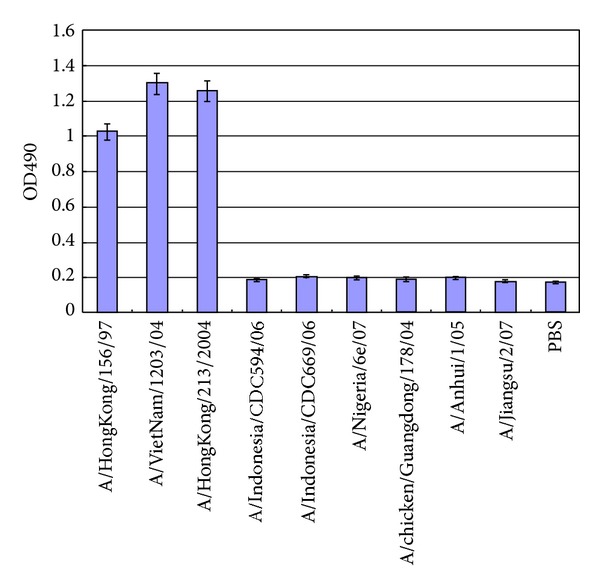
Reactivity against different H5 strains with 11G12 in AC-ELISA. 100 uL of each virus at 2^8^ HA titer was tested in the ELISA.

11G12 was tested in IFA, HI, ELISA, and virus neutralization against various H5 strains besides VN1203. As shown in Figure 1, 11G12 can only detect VN1203 and HK156 infection in IFA without any positive signals in MDCK cells infected with either CDC669 or Anhui strains. The results were confirmed in AC-ELISA (Figure 2). 11G12 can detect VN1203, HK156, and HK213 strains without any cross reaction to the rest of H5 strains tested. In HI and VN test (Table 1), 11G12 succeeded in inhibiting VN1203, HK156, and HK213; all of which belong to either clade 0 or 1, while 11G12 failed to neutralize any virus strain from other clades tested.

### 3.3. Specific Quantify VN1203 Expression in Trivalent H5 Vaccines

**Figure 3:**
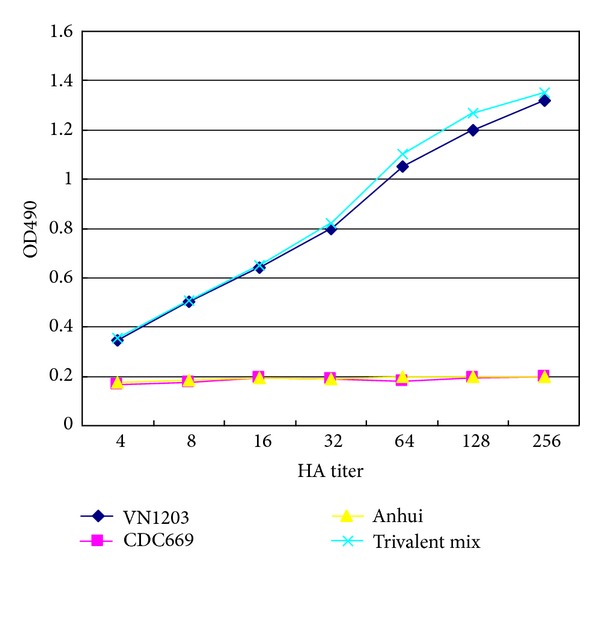
Binding curves with Mab 11G12 and monovalent and trivalent H5 mix.

A trivalent H5 vaccine was made from the mixture of H5 viruses including A/VietNam/1203/2004 (VN1203), A/Indonesia/CDC669/06, and A/Anhui/1/05. The three strains were individually tested in AC-ELISA to determine the binding curve with 11G12. AC-ELISA was further performed to identify and quantify VN1203 H5 expression in the trivalent mixture. As shown in Figure 3, 11G12 showed gradient reactivity against monovalent VN1203 at different HA titers, while negative signal was obtained against the rest two strains at all HA titers. The binding curve with trivalent H5 mix matched the one with mono-VN1203 at the same HA titers based on 11G12. At the meantime, the expression levels of Indonesia and Anhui H5 were quantified with the other two strain specific Mabs in the same method (data not shown).

### 3.4. Epitope Mapping for 11G12

Since Mab 11G12 is able to neutralize VN1203 H5N1 virus, the amino acids involved in forming the epitopes of Mab 11G12 were analyzed using selection of neutralization escape mutants. Point mutations in VN1203 H5 which abolish the neutralization by Mab 11G12 were identified. Sequencing of the complete HA genes isolated from 16 escape variants to 11G12 was performed. Six mutants were identified to carry single point mutations at amino acid positions 152 (Lys to Gln). Five strains mutate at 155 (Ser to Ile) and the other 5 strains at 189 (Lys to Arg) (excluding signal peptide) (Table 2).

## 4. Discussion

Generated from mice immunized with VN1203 H5N1 virus, 11G12 is able to recognize specifically H5 viruses from clade 0 and 1.0. The specific reactivity was observed in IFA, ELISA, and virus neutralization. 11G12 presented strong positive neutralizing reaction with all the tested viruses of clade 0 and 1.0, while 11G12 cannot react and neutralize any tested viruses from other clades. Mab 11G12 recognizes a neutralizing epitope containing at least 152Lys, 155Ser, and 189Lys on H5 hemagglutinin. The combination of three amino acids at these sites is mainly found in H5 influenza viruses from clade 0 and 1.0. These findings indicate that 11G12 recognizes a neutralizing epitope existing in early H5 strains, such as strains of clade 0 and 1.0. With mutations occurring in the antigenic structure during evolution, the affinity between 11G12 and the epitope was abolished finally, indicating that antigenic drift does cause virus evasion from the immunity elicited by vaccination with early strains. 

Mab 11G12 presents the binding preference to the antigenic structures belonging to these H5N1 strains of clade 0 and 1.0. This finding not only provides a solid evidence for antigenic drift, but also emphasizes the current needs for a universal H5 vaccine, which evokes an application of 11G12 in vaccine production. The specificity of Mab 11G12 allows it to identify and quantify VN1203 HA expression specifically in the trivalent H5 vaccine. The trivalent H5 universal vaccine is based on three types of H5s from different clades, different years, and different countries. The universal vaccine formulation can be produced based on three H5N1 influenza viruses mixed. Besides, the H5 combination can be expressed in any other vaccine production platform, such as baculovirus [18] and Modified vaccinia Ankara. For the maximal efficacy of the trivalent vaccine, it is important to keep the three H5s at the same proportion in the vaccine formulation. Thus, Mab 11G12 serves as an ideal reagent to specifically determine VN1203 H5 expression. Together with other specific Mabs for the rest two H5s, Mab 11G12 contributes to the quality control in vaccine production. 

Besides, employment of 11G12 in H5 diagnosis provides ease in clade-specific detection. H5 positive samples identified in traditional H5 rapid tests, such as lateral flow, can be further tested with 11G12 whether they belong to clade 0 or 1.0. The tests could be finished within 30 minutes, which are more efficient and cost-effective than real time PCR. Since cross-clade protection may not be available from every H5 vaccine and treatment, it is important to identify the H5 clade infected and provide effective therapeutic Mabs accordingly, which can be humanized 11G12. Together with other clade-specific Mabs for the other H5s, Mab 11G12 serves as a fine reagent for H5 clade-specific diagnosis.

## 5. Conclusion

Mab 11G12 can specifically recognize and neutralize H5 type hemagglutinin from clade 1 and 0 without any cross-reaction to any other clades of H5N1 viruses. The specificity of Mab 11G12 not only allows it to identify and quantify VN1203 HA expression specifically in a universal trivalent H5 vaccine, but also gives a solid evidence for the phenomenon of antigenic drift.

## Figures and Tables

**Table 1:** HI and virus neutralization activity of Mab 11G12^a^.

Virus	Clade	HI titer^b^	VN titer^c^
A/HongKong/156/97	0	112	160
A/VietNam/1203/04	1.0	256	320
A/HongKong/213/2004	1.0	256	320
A/Indonesia/CDC594/06	2.1.2	<4	<10
A/Indonesia/CDC669/06	2.1.3	<4	<10
A/Nigeria/6e/07	2.2	<4	<10
A/chicken/Guangdong/178/04	2.2	<4	<10
A/Anhui/1/05	2.3	<4	<10
A/Jiangsu/2/07	2.3	<4	<10
A/goose/Guiyang/337/06	4	<4	<10
A/chicken/Shanxi/2/06	7	<4	<10
A/chicken/Henan/12/04	8	<4	<10

^a^Concentration of MAb at 0.5 mg/mL.

^b^4 HA unit of each virus strain used for HI.

^c^One hundred TCID50 of each virus strain used for microneutralization assays.

**Table 2:** Neutralizing epitopes of H5 HA using 11G12 by escape mutations.

Parental virus	Nucleotide	Nucleotide change	Amino acid	Amino acid change
A/VietNam/1203/04	454	A to C	152	Lys to Gln
	464	G to T	155	Ser to Ile
	566	A to T	189	Lys to Met

## References

[1] Sambhara S, Poland GA (2010). H5N1 avian influenza: preventive and therapeutic strategies against a pandemic. *Annual Review of Medicine*.

[2] Lipatov AS, Smirnov YA, Kaverin NV, Webster RG (2005). Evolution of avian influenza viruses H5N1 (1997–2004) in southern and south-eastern Asia. *Voprosy Virusologii*.

[3] Yamada T, Dautry A, Walport M (2008). Ready for avian flu?. *Nature*.

[4] He F, Du Q, Ho Y, Kwang J (2009). Immunohistochemical detection of Influenza virus infection in formalin-fixed tissues with anti-H5 monoclonal antibody recognizing FFWTILKP. *Journal of Virological Methods*.

[5] Horimoto T, Fukuda N, Iwatsuki-Horimoto K (2004). Antigenic differences between H5N1 human influenza viruses isolated in 1997 and 2003. *Journal of Veterinary Medical Science*.

[6] Webster RG, Govorkova EA (2006). H5N1 influenza—continuing evolution and spread. *New England Journal of Medicine*.

[7] Yang P, Duan Y, Zhang P (2012). Multiple-clade H5N1 influenza split vaccine elicits broad cross protection against lethal influenza virus challenge in mice by intranasal vaccination. *PLoS ONE*.

[8] Yen HL, Aldridge JR, Boon ACM (2009). Changes in H5N1 influenza virus hemagglutinin receptor binding domain affect systemic spread. *Proceedings of the National Academy of Sciences of the United States of America*.

[9] Yen HL, Monto AS, Webster RG, Govorkova EA (2005). Virulence may determine the necessary duration and dosage of oseltamivir treatment for highly pathogenic A/Vietnam/1203/04 influenza virus in mice. *Journal of Infectious Diseases*.

[10] Chen GL, Subbarao K (2009). Attacking the flu: neutralizing antibodies may lead to “universal” vaccine. *Nature Medicine*.

[11] Lam TTY, Hon CC, Pybus OG (2008). Evolutionary and transmission dynamics of reassortant H5N1 influenza virus in Indonesia. *PLoS Pathogens*.

[12] Russell CA, Jones TC, Barr IG (2008). Influenza vaccine strain selection and recent studies on the global migration of seasonal influenza viruses. *Vaccine*.

[13] Prabakaran M, He F, Meng T (2010). Neutralizing epitopes of influenza virus hemagglutinin: target for the development of a universal vaccine against H5N1 lineages. *Journal of Virology*.

[14] Ho HT, Qian HL, He F (2009). Rapid detection of H5N1 subtype influenza viruses by antigen capture enzyme-linked immunosorbent assay using H5- And N1-specific monoclonal antibodies. *Clinical and Vaccine Immunology*.

[15] Abdel-Ghafar AN, Chotpitayasunondh T, Gao Z (2008). Update on avian influenza A (H5N1) virus infection in humans. *New England Journal of Medicine*.

[16] He Q, Velumani S, Du Q (2007). Detection of H5 avian influenza viruses by antigen-capture enzyme-linked immunosorbent assay using H5-specific monoclonal antibody. *Clinical and Vaccine Immunology*.

[17] Webster RG, Kawaoka Y, Taylor J, Weinberg R, Paoletti E (1991). Efficacy of nucleoprotein and haemagglutinin antigens expressed in fowlpox virus as vaccine for influenza in chickens. *Vaccine*.

[18] Prabakaran M, Velumani S, He F (2008). Protective immunity against influenza H5N1 virus challenge in mice by intranasal co-administration of baculovirus surface-displayed HA and recombinant CTB as an adjuvant. *Virology*.

[19] Kaverin NV, Rudneva IA, Govorkova EA (2007). Epitope mapping of the hemagglutinin molecule of a highly pathogenic H5N1 influenza virus by using monoclonal antibodies. *Journal of Virology*.

[20] Kaverin NV, Rudneva IA, Ilyushina NA (2002). Structure of antigenetic sites on the haeomagglutinin molecule of H5 avian influenza virus and phenotypic variation of escape mutants. *Journal of General Virology*.

